# High-throughput computation and evaluation of raman spectra

**DOI:** 10.1038/s41597-019-0138-y

**Published:** 2019-07-26

**Authors:** Qiaohao Liang, Shyam Dwaraknath, Kristin A. Persson

**Affiliations:** 1 Department of Materials Science and Engineering, University of California, Berkeley, California 94720 USA; 2 Lawrence Berkeley National Laboratory, Berkeley, California 94720 USA

**Keywords:** Computational methods, Electronic structure, Density functional theory

## Abstract

Raman spectroscopy is used ubiquitously in the characterization of condensed materials, spanning from biomaterials, minerals to polymers, as it provides a unique fingerprint of local bonding and environment. In this work, we design and demonstrate a robust, automatic computational workflow for Raman spectra that employs first-principle calculations based on density functional perturbation theory. A set of computational results are compared to Raman spectra obtained from established experimental databases to estimate the accuracy of the calculated properties across chemical systems and structures. Details regarding the computational methodology and technical validation are presented along with the format of our publicly available data record.

## Abstract (summary)

**Table:** 

Design Type(s)	modeling and simulation objective • database creation objective • data validation objective • chemical structure classification objective
Measurement Type(s)	Raman spectrum
Technology Type(s)	ab initio quantum chemistry computational method
Factor Type(s)	
Sample Characteristic(s)

Machine-accessible metadata file describing the reported data (ISA-Tab format)

## Background & Summary

Raman spectroscopy is an effective method for obtaining vibrational frequencies and local chemical bonding information in condensed materials. Due to its relative quick and non-destructive analytical nature, it is widely used in the analysis of crystalline materials and polymers - extending even into bio-medical materials and pharmaceuticals^1–3^, with advanced technologies and portable devices developed over the years. Indispensable for a complete vibrational spectrum observation, Raman spectroscopy has established an unique role in obtaining “fingerprints” for modern material science characterization. The increase in open access of experimentally measured Raman spectra databases, such as the RRUFF^4^ Project and Bio-Rad’s KnowItAll Raman Spectral Library, has incurred increased popularity. These resources now contain thousands of Raman spectral data of materials ranging from minerals, semiconductors to prescription drugs, which greatly facilitates the referencing process and identification of novel materials.

Compared to an experimental Raman database, a computational Raman Spectrum database from ab initio electronic structure calculations has many advantages^5–7^. Cheaper and more abundant computing resources enable thousands of calculations for a larger number of materials in an automated fashion, significantly reducing human effort. Systematic calculations result in a uniform data type, enabling accelerated classification of modes, discovery of correlations, and screening of useful materials. However, despite these advantages, there has been limited effort in constructing a computational Raman Spectrum database or a systematic workflow for its development. The WURM Project^8^ was an early prototype, but has shown little to no activity since 2012.

In this paper, we develop a computational workflow to calculate Raman spectra using ab initio density functional perturbation theory (DFPT): an accessible and fairly accurate tool for describing lattice dynamics^9^. Following the development of the dielectric tensor components in *Petousis et al*.^10^, computed directly using DFPT as implemented in VASP, the Raman tensors of 55 inorganic compounds are derived. They are then used to generate the Raman spectra data reported, which is benchmarked against experimental Raman spectra. These Raman spectra are integrated into the Materials Project (MP)^11^, a free, online database with computed properties to enable accelerated materials design. While Raman spectra are still computationally expensive, this work establishes a systematic workflow and associated benchmarking of numerical parameters for automated Raman spectra calculation that can be utilized by the community at large, and help increase the availability of reference Raman spectra for a wide variety of structures and chemical systems.

## Methods

### Theory and definitions

In Raman spectroscopy measurements of crystalline systems, incident laser photons with frequency *ω*_*L*_ interact with lattice vibrations, which can be described in the form of phonons. The inelastically scattered photons either exhibit a decrease or increase in frequency, resulting in symmetric Stokes or Anti-Stokes shifts, respectively. The recorded spectrum shows the intensity of the scattered light as a function of its frequency change, often converted to wavenumbers *v* in units of cm^−1^, known as Raman shifts. The Raman spectrum therefore provides a “fingerprint” description of the normal modes in the crystal through the comparison of incident and scattered photon frequencies. In our work, a normal mode’s wavenumber *v*_*mode*_ is directly obtained through a DFPT phonon calculations. The intensity of the mode *i* with polarization along *β* and electric field along *γ* is represented^12–15^ as

1 $$\begin{array}{l}{I}_{i\beta \gamma }=\frac{2\pi h{({\omega }_{L}-{\omega }_{i})}^{4}}{{c}^{4}{\omega }_{i}}[n({\omega }_{i})+1]{\alpha }_{i\beta \gamma }^{2}\end{array}$$

where *ω*_*i*_ is the vibrational mode frequency and *ω*_*L*_, the laser frequency, is usually arbitrarily set in computational methods since the shift in photon frequency is independent of incident photon frequency due to the nature of the interaction. $n({\omega }_{i})+1={\left(1-{e}^{\frac{-h{\omega }_{i}}{{k}_{B}T}}\right)}^{-1}$ is the Bose occupation number that is dependent on both temperature and frequency. In order to accommodate the most common experimental conditions, we set T = 300 K and *ω*_*L*_ to the wavenumber corresponding to a 532 nm wavelength laser. For each mode, the Raman tensor^12,16^

2 $$\begin{array}{l}{\alpha }_{i\beta \gamma }=\frac{\sqrt{{\rm{\Omega }}}}{4\pi }\sum _{ni\gamma }\,\frac{\partial {\varepsilon }_{\beta \gamma }}{\partial {u}_{in\nu }}{e}_{in\gamma }{m}_{n}^{-\frac{1}{2}}\end{array}$$

is obtained from the finite difference derivative of the dielectric tensor, *ε*_*βγ*_, with respect to the displacement *u*_*inv*_. *m*_*n*_ is the mass of atom n, *ν* the direction of displacement, Ω the unit cell volume, and *e*_*inγ*_ the eigenvector of the dynamical matrix. More specifically, the central difference scheme is employed as atoms are moved independently with displacement of 0.005 Å in both the positive (+) and negative (−) directions to calculate the differential of dielectric tensor with respect to displacement. However, the intensity expression in Eq. (1) is ideal for single crystals. Quite often experimental data use polycrystaline mineral specimens or powdered samples, in which case the intensity must be averaged over all possible orientations of the crystals which is accomplished by separating the total intensity *I* = *I*_⊥_ + *I*_||_^17–19^ into the depolarized light intensity

3 $$\begin{array}{l}{I}_{\perp } \sim {({\omega }_{L}-{\omega }_{i})}^{4}\frac{n({\omega }_{i})+1}{30{\omega }_{i}}[5{G}_{i,1}+3{G}_{i,2}]\end{array}$$

and the polarized light intensity

4 $$\begin{array}{l}{I}_{| | } \sim {({\omega }_{L}-{\omega }_{i})}^{4}\frac{n({\omega }_{i})+1}{30{\omega }_{i}}[10{G}_{i,0}+4{G}_{i,1}]\end{array}$$

where the rotation invariants^17,19^ are defined as

5 $$\begin{array}{l}{G}_{i,0}=\frac{1}{3}{({\alpha }_{ixx}+{\alpha }_{iyy}+{\alpha }_{izz})}^{2}\end{array}$$

6 $$\begin{array}{l}{G}_{i,1}=\frac{1}{2}[{({\alpha }_{ixy}-{\alpha }_{iyx})}^{2}+{({\alpha }_{ixz}-{\alpha }_{izx})}^{2}+{({\alpha }_{izy}-{\alpha }_{iyz})}^{2}]\end{array}$$

7 $$\begin{array}{lll}{G}_{i,2} & = & \frac{1}{2}[{({\alpha }_{ixy}+{\alpha }_{iyx})}^{2}+{({\alpha }_{ixz}+{\alpha }_{izx})}^{2}+{({\alpha }_{izy}+{\alpha }_{iyz})}^{2}]\\  &  & +\,\frac{1}{3}[{({\alpha }_{ixx}-{\alpha }_{iyy})}^{2}+{({\alpha }_{ixx}-{\alpha }_{izz})}^{2}+{({\alpha }_{izz}-{\alpha }_{iyy})}^{2}]\end{array}$$

For the Raman spectra of each compound, the intensities of each mode are normalized by setting the max intensity in the spectra to 100 and then scaling other mode intensities accordingly.

### Computational workflow

**Fig. 1:**
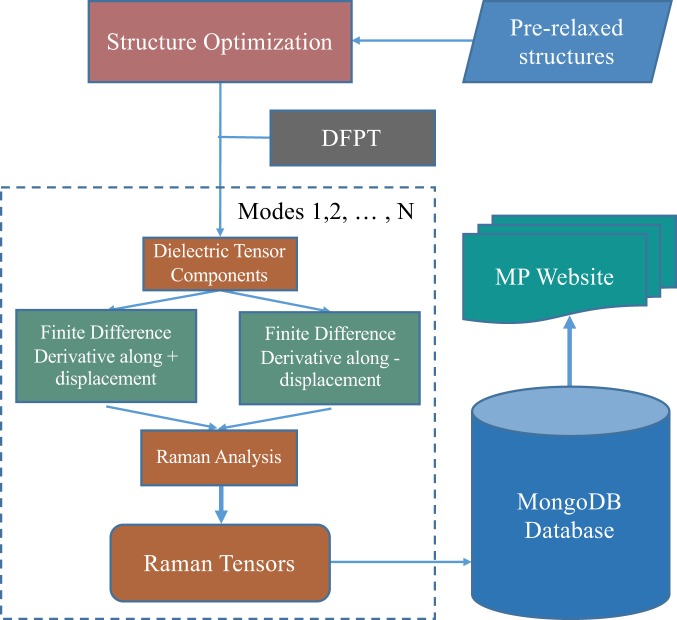
Computational workflow scheme used to calculate the Raman Tensors.

The workflow designed for automatic Raman Spectrum calculations is outlined in Fig. 1. The relaxed material structures are obtained from the Materials Project Database^11,20,21^ and serve as input structures to the following DFPT simulation. For all calculations of vibrational normal modes, DFPT simulations were run using the Vienna Ab-Initio Simulation Package, also known as VASP^22–25^, using the GGA/PBE^26^ + U^27,28^ exchange-correlation functional and projector augmented wave (PAW) pseudopotentials^29,30^. The k-point density was set at 3,000 per reciprocal atom and the plane wave energy cut-off at 600 eV, same as those in the calculation of dielectric tensors^10^. The structure is then displaced along the calculated normal mode eigenvectors for dielectric tensor calculations. Raman susceptibility tensors are calculated from the finite difference derivative of the dielectric tensor as shown in Eq. (2) and the collection of computational Raman analysis data is stored with all associated metadata from the Materials Project.

## Data Records

### Computational data record

The computational data is stored in a lightweight data-interchange format, and the JSON document can be downloaded directly from the Figshare repository^31^. The nested key/value pairs can be navigated using Tables 1 and 2. The document includes the original structure data from the Materials Project, normal modes information, tensors, and calculated intensities.

**Table 1:** Description of the nested JSON structure for the ‘Computational Data’.

Key	Datatype	Length/Size	Description
perturbed_structures	dictionary	nmodes	Perturbed crystal structures for Raman analysis
nmodes	number	—	Number of vibrational modes
eigenvals	array	nmodes	List of eigenvalues
eigenvecs	array	nmodes	List of eigenvectors
dielectric_tensor	array	nmodes	List of Dielectric tensors
raman_tensor	array	nmodes	List of Raman tensors
wavenumbers	array	nmodes	List of Raman shifts cm^−1^
intensity	array	nmodes	List of calculated intensities post-normalization
metadata	dictionary	7	Crystal structure before perturbation. Refer to Table 2.

**Table 2:** Description of the nested JSON structure for ‘metadata’.

Key	Datatype	Description
mp_id	string	Materials Project ID
formula	string	Chemical Formula
structure	dictionary	Unperturbed crystal structure from MP
point_group	string	Hermann-Mauguin Notation
space_group	number	Space_group number defined by International Union of Crystallography
crystal_system	string	Crystal system
nsites	number	Number of atoms in primitive cells

### Experimental data record

The experimental data extracted from the RRUFF database is provided in a separate JSON document with nested structure as shown in Table 3. The file includes mineral type, peak locations, intensity values, RRUFF_id, and matched Materials Project IDs. To ensure identical structures, each mineral is matched to a compound from the Materials Project by fitting Crystallographic Information Files (CIF) using pymatgen tools or manually referencing to mineralogy data^32^. The RRUFF spectra data and all CIF format files used are included in the Figshare repository^31^ for increased transparency and ease in reproduction of the benchmarking results.

**Table 3:** Description of the nested JSON structure for extracted ‘Experimental Data’.

Key	Datatype	Description
type	array	Mineral Type(s)
formula	string	Chemical Formula and Mineral Name
RRUFF_id	string	ID from RRUFF database
noise	number	Background noise intensity (before normalization)
start	number	Smallest wavenumber value recorded in the Raman spectra
wavenumbers	array	List of peak locations
intensities	array	List of peak intensities (before normalization)
mp_id	string	Matched ID from the Materials Project

## Technical Validation

Unlike evaluating the accuracy of scalar material properties, comparison of the Raman spectra requires a matching process between the calculated vibrational modes and experimental Raman spectra peaks from the RRUFF database^4^. Hence, a linearly modelled cost metric, *u* = *w*_1_(*v*_*mode*_ − *v*_*peak*_) + *w*_2_(*I*_*mode*_ − *I*_*peak*_) = *w*_1_Δ*v* + *w*_2_Δ*I*, considering the proximity in both wavenumber and normalized intensity is used in the matching process: within the same compound, each of the computed vibrational modes is paired with a corresponding experimental peak that minimizes the overall cost. To ensure the matching process produces more accurate results, we process the calculated Raman spectra data in the following two steps.

First, for each compound’s computed Raman spectra, a signal to noise threshold of 0.356% is used to remove vibrational modes with experimentally undetectable intensities. This threshold is the average noise intensity to maximum intensity obtained from reference experimental data. Second, for each compound, the low wavenumber region is removed from the computational Raman spectrum for both experimental and computational reasons. From an experimental point of view, the RRUFF database records different initial probing locations for different compounds’ Raman spectra, which presents a noticeable challenge for the peak matching process. The average distance between the starting location of RRUFF Raman spectra and its first peak location is 113 cm^−1^, which provides a location reference for the computational Raman spectra of each compound. From a computational perspective, DFPT tend to exhibit larger errors at lower wavenumbers; these modes correspond to long range oscillations that are difficult to compute within limited size of periodic unit cells. For example, these low wavenumber modes, corresponding to phonon wavelengths on the orders of microns, can be influenced by long range features such as defects, dislocations and grain boundaries that are not captured in the simulations of perfectly periodic, single crystal structures.

**Fig. 2:**
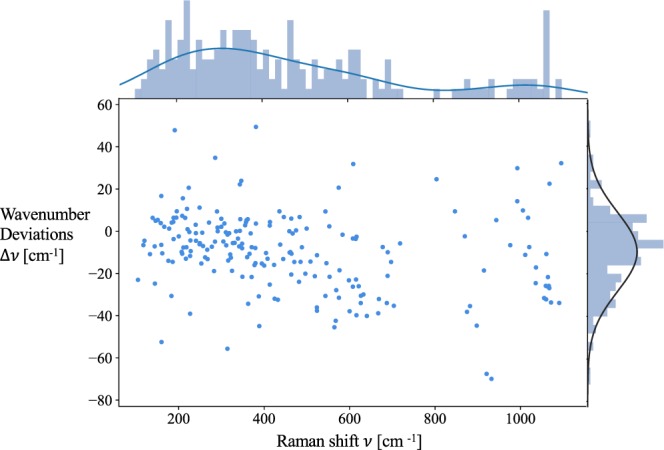
Plot of wavenumber deviations Δ*v* of computed modes versus their wavenumbers. Each data point belongs to a matched pair of calculated phonon mode and experimental peak. The x axis shows the wavenumbers of phonon modes, *v* = *v*_*mode*_, while the y axis shows how much these wavenumbers of phonon modes differ from those of their matched experimental peaks, namely Δ*v* = *v*_*mode*_ − *v*_*peak*_. The Gaussian kernel density estimates of *v* and the normal distribution of Δ*v* are shown respectively on the x and y axis.

**Fig. 3:**
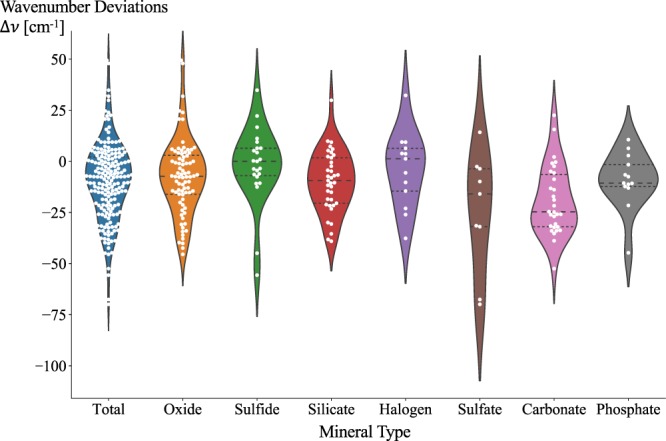
Distribution of wavenumber deviations Δ*v* by mineral type. The violin outlines are Gaussian kernel density estimates of the data points in the middle. The quartiles are labelled with dashed lines.

The data consists of 55 compounds and 205 pairs of matched peaks/modes, where the total calculation cost amounted to 9.5 Million CPU hours. The accuracy of the calculated phonon modes’ wavenumber values *v*_*mode*_ is represented in Fig. 2, where the compounds range from simple oxides, sulfides to carbonates. The average wavenumber deviation Δ*v* for all the compounds is −9.66 cm^−1^ with a standard deviation of 18.58 cm^−1^, which indicates that our calculations tend to slightly underestimate the Raman peak locations. With a mean absolute relative error (MARE) of −2%, we conclude that our test set is in reasonably good agreement with the experimental data available. In Fig. 3, violin plots are used to depict the wavenumber deviations grouped by mineral type. The oxide family consists 40% of the data, and shows weak underestimation of peak locations with average absolute wavenumber deviation of −7.5 cm^−1^. The sulfate and carbonate families exhibit the largest wavenumber deviations. While the sulfate family’s larger deviation is more likely due to the limited number of sulfate compounds used in this work, the carbonate family exhibits more abundant data for distribution analysis, showcasing in an average wavenumber deviation of −19.3 cm^−1^. Most notably, we observe large wavenumber deviations of carbonates’ modes at Raman shifts around 1000 cm^−1^. These modes are all generated from the vibrations of oxygen anions, which could be affected by small (unknown) amounts of water in mineralogical samples, which are not accounted for in the calculations.

**Fig. 4:**
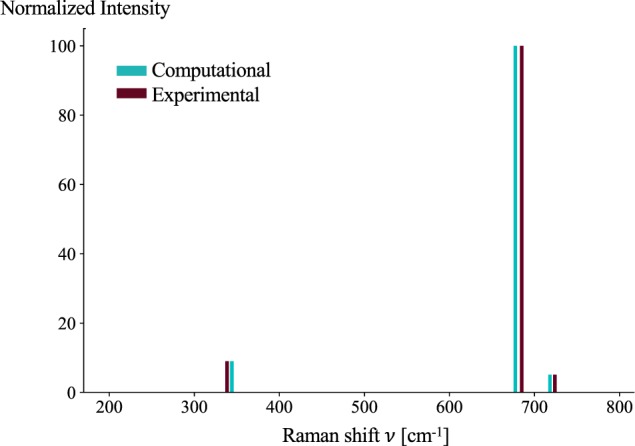
The computational and experimental Raman spectra of BeO (Bromellite). The direct comparison of computational and experimental Raman spectra shows that even when the peaks and modes are well paired, intensity deviation can still be observed.

The intensity deviations do not show a clear bias. Most often, as seen in Fig. 4, carefully tuned weights *w*_1_ and *w*_2_ can yield well paired peaks and modes, yet discrepancies in intensity still remain. It should be noted that our structures from MP assume a perfectly ordered bulk crystal at 0 K, and therefore extrinsic factors associated with temperature, pressure, defects, and phonon anharmonicity are not considered. These effects on the dielectric tensors are described in detail in Petousis *et al*.^33^, which carries over to the Raman tensors and eventually affects the calculated intensities. Interfaces and defects in particular are expected to be a strong scattering center for phonons which could account for much of the intensity variation as the phonon population deviates from a simple Bose-Einstein distribution.

**Fig. 5:**
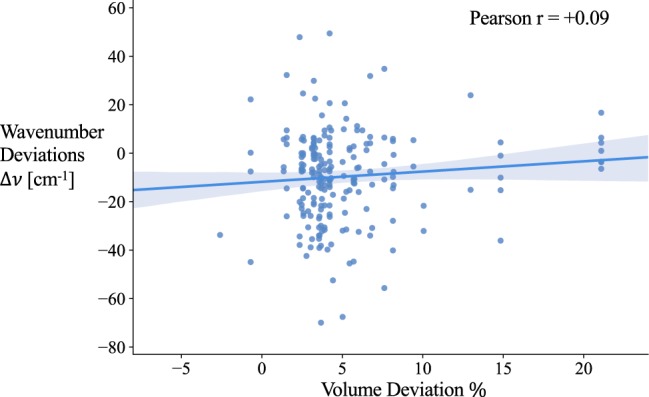
Plot of wavenumber deviation Δ*v* of computed modes versus volume deviation % of the compounds they belong to. The volume deviation % of each compound is calculated as $\frac{{V}_{MP}-{V}_{RRUFF}}{{V}_{MP}}\cdot 100 \%$.

Besides the extrinsic effects mentioned above, there are other factors that could contribute to discrepancies between computational and experimental values. One such effect is that of the use of the GGA/PBE exchange-correlation function, which has been shown to exhibit lattice under binding^34^, yielding larger bond lengths, lattice parameters and unit cell volumes of the relaxed structures. We investigated the correlation between the unit cell volume expansions % and Raman peaks’ wavenumber discrepancies (cm^−1^) to determine if the GGA/PBE known under-binding causes a systematic and significant deviation of the computed Raman data. Despite an average volume expansion of 4.94%, the weak correlation between the two (+0.09), as seen in Fig. 5, suggested that the effects of GGA/PBE is not significant in this work. Therefore, we do not attribute the observed wavenumber discrepancies to the use of GGA/PBE exchange correlation function. Other salient differences between computational assumptions and experimental conditions may also contribute to the discrepancies. For example, the applied rotation invariant intensity equation is an average result considering every possible scattering geometry, and therefore assumes a powdered or isotropic poly-crystalline sample. However, the quality of mineral samples do not always satisfy these ideal conditions, which may allow specific sample orientations or laser polarization directions in experimental measurements to create discrepancies in Raman spectra.

## Usage Notes

An automatic workflow for ab initio electronic structure Raman spectra is expected to be of interest to the broad community of materials science and chemistry. We expect this growing dataset to be used in the understanding of Raman characterization and provide a reference for experimentally measured Raman data. Furthermore, our work opens up opportunities in the development of a data intensive approaches. For example, the application of machine learning techniques on existing spectra may be utilized to enhance the data set as well as to improve the peak matching process. Such work provide the basis for quick identification by screening through compounds on the Materials Project website, comparing their computational Raman spectra data to the reference experimental counterpart. Still, we emphasize that the computational cost for developing this database is rather significant. While this work illustrates the promise of a computational Raman spectra database, further work is necessary to reduce the computational cost to access truly large data sets needed for machine learning approaches.

## ISA-Tab metadata file

Download metadata file

## Data Availability

The proprietary VASP-code is primarily used in the DFPT calculations. The processing and modifications of the simulations were implemented using Pymatgen^20^ and FireWorks^35^. Pymatgen (Python Materials Genomics) is an open-source Python library under Massachusetts Instrutute of Technology (MIT) license for materials analysis. The workflow shown in Fig. 1 is implemented using FireWorks in Atomate^36^, which stores, executes, and manages calculation workflows and is free to public through Atomate’s Github site under a modified GNU General Public License.
